# Difference-based ridge-type estimator of parameters in restricted partial linear model with correlated errors

**DOI:** 10.1186/s40064-016-1814-4

**Published:** 2016-02-25

**Authors:** Jibo Wu

**Affiliations:** School of Mathematics and Finances, Chongqing University of Arts and Sciences, Chongqing, 402160 China; Key Laboratory of Group & Graph Theories and Applications, Chongqing University of Arts and Sciences, Chongqing, 402160 China

**Keywords:** Differencing estimator, Multicollinearity, Linear equality restrictions, Partial linear model, Ridge estimator, Primary 62J05, 62J07

## Abstract

In this article, a generalized difference-based ridge estimator is proposed for the vector parameter in a partial linear model when the errors are dependent. It is supposed that some additional linear constraints may hold to the whole parameter space. Its mean-squared error matrix is compared with the generalized restricted difference-based estimator. Finally, the performance of the new estimator is explained by a simulation study and a numerical example.

## Background

Consider the following partially linear model

1 $$\begin{aligned} y_i = x'_i\beta + f(t_i)+ \varepsilon _i,\quad i=1,\ldots ,n \end{aligned}$$

where $y_i's$ are observations, $x_i'=(x_{i1},\ldots ,x_{ip})$ and $x_1,\ldots ,x_n$ are known p-dimensional with $p\le n$. $t_i's$ are values of an extra univariate variable such as the time at which the observation is made, $\beta =(\beta _1,\ldots ,\beta _p)'$ is an unknown parameter vector. $f(\cdot )$ is an unknown smooth function, and $\varepsilon _i's$ are random errors supposed to be *i*.*i*.*d*. $N(0,\sigma ^2)$ distributed.

Use matrix vector notation, model (1) can be written as follows:

2 $$\begin{aligned} y=X\beta +f+\varepsilon \end{aligned}$$

where $$y=(y_1,\ldots ,y_n)'$$, $$X'=(x_1,\ldots ,x_n)$$, $$f=\left[ f(t_1),\ldots ,f(t_n)\right] '$$ and $$\varepsilon =(\varepsilon _1,\ldots ,\varepsilon _n)'$$.

Since partially linear model has parametric and nonparametric components, and it is more flexible than linear model, many authors have been studied it, such as Ahn and Powell (1993), Wang et al. (2007).

In model (2), Yatchew (1997) mainly studied the estimation of the linear component and used differencing to eliminate bias induced from the presence of the nonparametric component. Wang et al (2007) presented higher-order differences for optimal efficiency in estimating the linear part by using a special class of difference sequences.

In this article we will use the ridge regression concept that was presented by Hoerl and Kennard (1970) to overcome the multicollinearity in regression problem. Multicollinearity is denoted as the existence of nearly linear dependency among column vectors of the design matrix *X* in the linear model $y=X\beta +\epsilon$, where *y* is a $n\times 1$vector of observed responses, *X* is the observed matrix of independent variables of dimension $n\times p$, assumed to have full rank *p*, $\beta$ is an unknown parameter, $\epsilon$ is an error vector with $E(\epsilon )=0, E(\epsilon \epsilon ')=\sigma ^{2}I_{p}$. Multicollinearity may lead to wide confidence intervals for individual parameters may produce estimates with wrong signs, etc.

The condition number is a measure of the presence of multicollinearity. The condition number of the matrix *X* present some information about the existence of multicollinearity, however it does not illustrate the structure of the linear dependency among the column vectors $X_{1}, X_{2}, \ldots , X_{n}$. The best way of illustrating the existence and structure of multicollinearity is to see the eigenvalues of $X'X$. If $X'X$ is ill-conditioned with a large condition number a ridge regression estimator can be used to estimate $\beta$ [see e.g., Swamy et al. (1978); Sarkar (1992); Shi (2001); Zhong and Yang (2007); Zhang and Yang (2007); Tabakan and Akdeniz (2010); Akdeniz and Tabakan (2009); Roozbeh et al. (2010); Duran and Akdeniz (2012); Duran et al. (2012); Hu (2005) and Hu et al. (2015)]. In this paper, we will examine a biased estimation techniques to be followed when the matrix $X'X$ appears to be ill-conditioned in the partial linear model. We suppose that the condition number of the parameteric component is large explain that a biased estimation procedure is desirable.

The rest of the paper is organized as follows. In section “The model and differencing-based estimator”, the model and differencing methodology are given. Section “Generalized difference-based ridge estimator” contains the definition of the generalized difference-based ridge estimator and some comparison results are given in section “[Sec Sec4]”. The results from section “[Sec Sec4]” are applied to a simulation study in section “Exemplary simulation” and a numerical example is given to illustrate the theoretical result in section “A numerical example”. Some conclusion remarks are given in section “Conclusions”.

## The model and differencing-based estimator

In this section we use a difference-based method to estimate the linear regression coefficient vector $\beta$. This method has been presented to remove the nonparametric component in the partially linear model by many authors (Yatchew 1997, 2000, 2003). Consider the following partially linear model

3 $$\begin{aligned} y=X\beta +f+\varepsilon \end{aligned}$$

where *f* is an unknown smooth function and has a bounded first derivative.

Now we present the differencing method. Let $d=(d_{0},\ldots ,d_{m})$ be a $m+1$ vector, where *m* is the order of differencing and $d_{0},\ldots ,d_{m}$ are differencing weights satisfying the conditions

4 $$\begin{aligned} \sum _{j=0}^{m}d_{j}=0,\quad \sum _{j=0}^{m}d_{j}^{2}=1 \end{aligned}$$

Now, we denote the $(n-m)\times n$ differencing matrix *D* whose elements satisfy Eq. (4) as follows:

5 $$\begin{aligned} D=\left( \begin{array}{cccccccc} d_{0}&{}d_{1}&{}\ldots &{}d_{m}&{}0&{}0&{}\ldots &{}0\\ 0&{}d_{0}&{}d_{1}&{}\ldots &{}d_{m}&{}0&{}\ldots &{}0\\ \ldots &{}\ldots &{} &{} &{} &{} &{} &{} \\ \ldots &{}\ldots &{} &{} &{} &{} &{} &{}\\ \ldots &{}\ldots &{} &{} &{} &{} &{} &{}\\ 0&{}0&{}\ldots &{}d_{1}&{}\ldots &{}d_{m}&{}0&{}0\\ 0&{}0&{}\ldots &{}d_{0}&{}d_{1}&{}\ldots &{}d_{m}&{}0\\ 0&{}0&{}\ldots &{}0&{}d_{0}&{}d_{1}&{}\ldots &{}d_{m}\\ \end{array} \right) \end{aligned}$$

This and related matrices are given, for example, in Yatchew (2003). Then we can use the differencing matrix to model (3), and this leads to direct estimation of the parametric effect. In particular, take

6 $$\begin{aligned} Dy=DX\beta +Df(t)+D\epsilon \end{aligned}$$

Since the data have been reordered so that the $X's$ are close, the application of the differencing matrix *D* in model (6) can remove the nonparametric effect in large samples (Yatchew 2003). This ingores the presence of *Df*(*t*). Thus, we may write Eq. (6) as

7 $$\begin{aligned} Dy \doteq DX\beta +D\epsilon \end{aligned}$$

or

8 $$\begin{aligned} \widetilde{y} \doteq \widetilde{X}\beta +\widetilde{\epsilon } \end{aligned}$$

where $$\widetilde{y}=Dy, \widetilde{X}=DX$$ and $$\widetilde{\epsilon }=D\epsilon$$.

So, we can see that $\widetilde{\epsilon }$ is a $n-m$ vector of disturbances distributed with $E(\widetilde{\epsilon })=0\quad \text {and} \quad E(\widetilde{\epsilon }\widetilde{\epsilon }')=\sigma ^{2}DD'$.

For arbitrary differencing coefficients satisfying Eq. (8), Yatchew (1997) defines a simple differencing estimator of the parameter $\beta$ in a partial linear model

9 $$\begin{aligned} \hat{\beta }=(\widetilde{X}'\widetilde{X})^{-1}\widetilde{X}'\widetilde{y} \end{aligned}$$

Hence, differencing allows one to perform inferences on $\beta$ as if there were no nonparametric component *f*() in the model (3) (Yatchew 2003). Once $\beta$ is estimated, a variety of nonparametric techniques could be applied to estimate *f*() as if $\beta$ were known.

In order to account for the parameter $\beta$ in Eq. (3), we propose the modified estimator of $\sigma ^{2}$, defined as

10 $$\begin{aligned} \hat{\sigma }^{2}=\frac{\widetilde{y}'(I-P)\widetilde{y}}{tr(D'(I-P)D)} \end{aligned}$$

where *P* is the projection matrix and defined as

11 $$\begin{aligned} P=\widetilde{X}(\widetilde{X}'\widetilde{X})^{-1}\widetilde{X}' \end{aligned}$$

## Generalized difference-based ridge estimator

In this section we discuss the following partially linear model:

12 $$\begin{aligned} y=X\beta +f+\varepsilon \end{aligned}$$

with $E(\varepsilon )=0$ and $E(\varepsilon '\varepsilon )=\sigma ^2V$. So using the method we proposed in section “The model and differencing-based estimator”, we have $\tilde{\varepsilon }=D\varepsilon$ is a $(n-m)$-vector of disturbances distributed with

13 $$\begin{aligned} E(\tilde{\varepsilon })=0 \quad \text {and} \quad E(\tilde{\varepsilon }'\tilde{\varepsilon })=\sigma ^2DVD'=\sigma ^2V_D \end{aligned}$$

where $V_D=DVD'\ne I_{n-m}$ is a known $(n-m)\times (n-m)$ symmetric positive definite matrix.

It is well known that adopting the linear model (12), the unbiased estimator of $\beta$ is the following generalized difference-based estimator given by

14 $$\begin{aligned} \hat{\beta }_{GD}=C_{D}^{-1}\widetilde{X}'V_{D}^{-1}\widetilde{y},\quad C_{D}=\widetilde{X}'V_{D}^{-1}\widetilde{X} \end{aligned}$$

and the modified estimator $\sigma ^{2}$,

15 $$\begin{aligned} \hat{\sigma }^{2}=\frac{\widetilde{y}'V_{D}^{-1/2}(I-P)V_{D}^{-1/2}\widetilde{y}}{tr(D'(I-P)D)} \end{aligned}$$

where *P* is the projection matrix and defined as

16 $$\begin{aligned} P=V_{D}^{-1/2}\widetilde{X}(\widetilde{X}'V_{D}^{-1}\widetilde{X})^{-1}\widetilde{X}'V_{D}^{-1/2} \end{aligned}$$

It is observed from Eq. (14) that the properties of the generalized difference-based estimator of $\beta$ depends heavily on the characteristics of the information matrix $C_{D}$. If the $C_{D}$ matrix is ill-conditioned, then the $\hat{\beta }_{GD}$ leads to large sampling variances. Moreover, some of the regression coefficients may be statistically insignificant with wrong sign and meaningful statistical inference becomes difficult for the researcher. As a remedy, we consider the linear constraint

17 $$\begin{aligned} R\beta =0 \end{aligned}$$

for a given $q\times p$ matrix *R* with rank $q< p$. Subject to the linear restriction (17), the generalized restricted difference-based estimator is given by

18 $$\begin{aligned} \hat{\beta }_{GRD}=\hat{\beta }_{GD}-C_{D}^{-1}R'(RC_{D}^{-1}R')^{-1}R\hat{\beta }_{GD} \end{aligned}$$

Define $W=C_{D}^{-1}-C_{D}^{-1}R'(RC_{D}^{-1}R')^{-1}RC_{D}^{-1}$, we obtain

19 $$\begin{aligned} \hat{\beta }_{GRD}=W\widetilde{X}'V_{D}^{-1}\widetilde{y} \end{aligned}$$

Now we propose a generalized difference-based ridge estimator, which is defined as

20 $$\begin{aligned} \hat{\beta }_{GRD}(k)=(kW+I)^{-1}\hat{\beta }_{GRD} \end{aligned}$$

where $$W=C_{D}^{-1}-C_{D}^{-1}R'(RC_{D}^{-1}R')^{-1}RC_{D}^{-1}$$ and $$k\ge 0$$.

Then, it is easy to see that $\hat{\beta }_{GRD}$ and $\hat{\beta }_{GRD}(k)$ are restricted with respect to $R\beta =0$. It is also clear that for $k=0$, we obtain $\hat{\beta }_{GRD}(0)=\hat{\beta }_{GRD}$.

## MSEM-superiority of the generalized difference-based ridge estimator $\hat{\beta }_{GRD}(k)$ over the the generalized restricted difference-based estimator $\hat{\beta }_{GRD}$

In this section, our aim is to examine the difference of the mean squared error matrices (MSEM) of two estimators $\hat{\beta }_{GRD}(k)$ and $\hat{\beta }_{GRD}$. Let $b^{*}$ be an estimator of $\beta$ in model $Y=X\beta +\epsilon$. The MSEM of $b^{*}$ is defined as

21 $$\begin{aligned} \text{ MSEM}(b^{*}, \beta )=E\left[ (b^{*}-\beta )(b^{*}-\beta )'\right] \end{aligned}$$

If we denote the covariance matrix of an estimator $b^{*}$ by $V(b^{*})$, then (21) is equivalent to

22 $$\begin{aligned} \text{ MSEM}(b^{*}, \beta )=\text{ Var}(b^{*}) +\left( bias(b^{*})\right) (bias(b^{*}))' \end{aligned}$$

where $bias(b^{*})=E(b^{*})-\beta$. The scalar valued mean square error MSE is given by $\text{ MSE}(b^{*}, \beta )=E\left[ (b^{*}-\beta )'(b^{*}-\beta )\right] \,=\,\text{ tr }[\text{ MSEM}(b^{*}, \beta )]$.

Using Eq. (20), we obtain

23 $$\begin{aligned} E(\hat{\beta }_{GRD}(k))=-k(kW+I)^{-1}W\beta \end{aligned}$$

and

24 $$\begin{aligned} \text{ Var}(\hat{\beta }_{GRD}(k))=\sigma ^{2}(kW+I)^{-1}W(kW+I)^{-1} \end{aligned}$$

Thus,

25 $$\begin{aligned} \text{ Var}(\hat{\beta }_{GRD})=\sigma ^{2}W \end{aligned}$$

Then, the difference $\text{ Var}(\hat{\beta }_{GRD})-\text{ Var}(\hat{\beta }_{GRD}(k))$ can be expressed as

26 $$\begin{aligned} \text{ Var}(\hat{\beta }_{GRD})-\text{ Var}(\hat{\beta }_{GRD}(k))=\sigma ^{2}(kW+I)^{-1}(k^{2}W^{3}+2kW^{2})(kW+I)^{-1} \end{aligned}$$

Since *W* is an nonnegative definite matrix [see Shi (2001)], we can conclude that $\text{ Var}(\hat{\beta }_{GRD})-\text{ Var}(\hat{\beta }_{GRD}(k))$ is an nonnegative definite matrix.

It is of interest to know under which conditions $\hat{\beta }_{GRD}(k)$ is better than $\hat{\beta }_{GRD}$. For this, we investigate the difference $\Delta = \text{ MSEM}(\hat{\beta }_{GRD}, \beta )-\text{ MSEM} (\hat{\beta }_{GRD}(k), \beta )$, when $\Delta$ is nonnegative definite matrix, $\hat{\beta }_{GRD}(k)$ is preferred to $\hat{\beta }_{GRD}$. Thus, for the MSE, of the generalized difference-based ridge estimator $\hat{\beta }_{GRD}(k)$, from (23) and (24), we obtain

27 $$\begin{aligned} \text{ MSEM}(\hat{\beta }_{GRD}(k), \beta )\,=\, & {} \text{ Var}(\hat{\beta }_{GRD}(k))+(bias(\hat{\beta }_{GRD}(k))(bias(\hat{\beta }_{GRD}(k)))' \nonumber \\\,=\, & {} \sigma ^{2}(kW+I)^{-1}W(kW+I)^{-1} \nonumber \\&+k^{2}(kW+I)^{-1}W\beta \beta 'W(kW+I)^{-1} \end{aligned}$$

Since $\hat{\beta }_{GRD}$ is unbiased estimator for $\beta$, we have

28 $$\begin{aligned} \text{ MSEM}(\hat{\beta }_{GRD},\beta )=\text{ Var}(\hat{\beta }_{GRD})=\sigma ^{2}W \end{aligned}$$

Now from (27) and (28), we may write the difference $\Delta = \text{ MSEM}(\hat{\beta }_{GRD}, \beta )-\text{ MSEM} (\hat{\beta }_{GRD}(k), \beta )$

29 $$\begin{aligned} \Delta\,=\, & {} \text{ MSEM }(\hat{\beta }_{GRD}, \beta )-\text{ MSEM } (\hat{\beta }_{GRD}(k),\beta )\nonumber \\\,=\, & {} \sigma ^{2}W-\sigma ^{2}(kW+I)^{-1}W(kW+I)^{-1} \nonumber \\&-k^{2}(kW+I)^{-1}W\beta \beta 'W(kW+I)^{-1} \nonumber \\\,=\, & {} (kW+I)^{-1}(\sigma ^{2}k^{2}W^{3}+2\sigma ^{2}kW^{2}-k^{2}W\beta \beta 'W)(kW+I)^{-1} \end{aligned}$$

Then, by (29), $\Delta = \text{ MSEM}(\hat{\beta }_{GRD}, \beta ) -\text{ MSEM} (\hat{\beta }_{GRD}(k), \beta )\ge 0$ if and only if $\sigma ^{2}k^{2}W^{3}+2\sigma ^{2}kW^{2}-k^{2}W\beta \beta 'W\ge 0$.

Then using Theorem (Farebrother 1976), we can conclude that if $k>0,\; \beta '\left( W+\frac{2}{k}I\right) ^{-1}\beta \le \sigma ^{2}$, then $\hat{\beta }_{GRD}(k)$ is preferred to $\hat{\beta }_{GRD}$.

### **Theorem 4.1**

*Consider the two estimator *$\hat{\beta }_{GRD}$* and *$\hat{\beta }_{GRD}(k)$* of *$\beta$*. Then the biased estimator *$\hat{\beta }_{GRD}(k)$*is MSEM-superior over the *$\hat{\beta }_{GRD}$* if*

30 $$\begin{aligned} \beta '\left( W+\frac{2}{k}I\right) ^{-1}\beta \le \sigma ^{2} \end{aligned}$$

*is satisfied.*

## Exemplary simulation

In this section, we study the MSE of the proposed estimator. Our sampling experiment consists of different combinations of *k* and *n*. In this paper, we simulate the response from the following model:

31 $$\begin{aligned} y=x_{1i}\beta _{1}+x_{2i}\beta _{2}+x_{3i}\beta _{3}+x_{4i}\beta _{4}+f(t_{i})+\epsilon _{i} \end{aligned}$$

where $i=1,\ldots ,n$, $\epsilon \sim (0,\sigma ^{2}V)$ which the elements of *V* is $v_{ij}=(0.1)^{|i-j|}$ and $\sigma =0.1$, $f(t_{i})=\sqrt{t_{i}(1-t_{i})}\sin \frac{2.1\pi }{t_{i}+0.05}$ that is called Doppler function for $t_{i}=(i-0.5)/n$ and for $i=1,\ldots ,n$, the explanatory variables are generated by the following equation (Liu 2003):

$$\begin{aligned} x_{ij}=(1-\gamma ^2)z_{ij}+\gamma z_{i(p+1)},\quad i=1,\ldots ,n,\ j=1,\ldots ,p \end{aligned}$$

where $z_{ij}$ and $z_{i(p+1)}$ are independent standard normal pseudo-random numbers and $\gamma$ is specified so that the correlation between any two explanatory variables is given by $\gamma ^2$. In this paper, we consider $n=200$ and $p=4$.

In this article we use a third-order differencing coefficients $d_{0} = 0.8502$, $d_{1}=-0.3832$, $d_{2}= -0.2809$, $d_{3}=-0.1942$ in which $m=3$. Now, we define the $(200-3)\times 200$ differencing matrix as follows:

32 $$\begin{aligned} D=\left( \begin{array}{cccccccc} d_{0}&{}d_{1}&{}\ldots &{}d_{m}&{}0&{}0&{}\ldots &{}0\\ 0&{}d_{0}&{}d_{1}&{}\ldots &{}d_{m}&{}0&{}\ldots &{}0\\ \ldots &{}\ldots &{} &{} &{} &{} &{} &{} \\ \ldots &{}\ldots &{} &{} &{} &{} &{} &{}\\ \ldots &{}\ldots &{} &{} &{} &{} &{} &{}\\ 0&{}0&{}\ldots &{}d_{1}&{}\ldots &{}d_{m}&{}0&{}0\\ 0&{}0&{}\ldots &{}d_{0}&{}d_{1}&{}\ldots &{}d_{m}&{}0\\ 0&{}0&{}\ldots &{}0&{}d_{0}&{}d_{1}&{}\ldots &{}d_{m}\\ \end{array} \right) \end{aligned}$$

For the linear restriction (17), the *R* is given as follows:

33 $$\begin{aligned} R=(1,-2,-2,-2) \end{aligned}$$

**Fig. 1:**
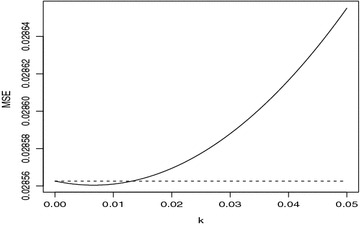
The estimated MSE of GRD and GRDR for $\gamma =0.8$

**Fig. 2:**
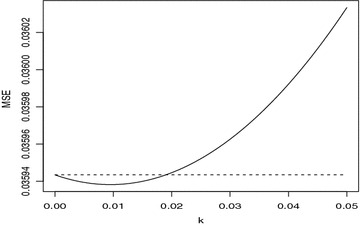
The estimated MSE of GRD and GRDR for $\gamma =0.85$

**Fig. 3:**
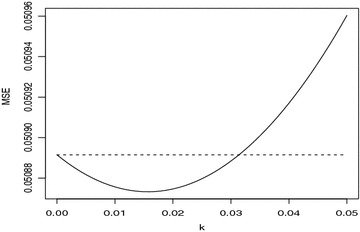
The estimated MSE of GRD and GRDR for $\gamma =0.9$

Let GRD define the generalized restricted difference-based estimator and GRDR define the generalized restricted difference-based ridge estimator and the estimated MSE of GRD and GRDR are given in Figs. 1, 2 and 3.

From Figs.  1 and  3, we see that we *k* is smaller, the new estimator is better than the generalized difference-based estimator in the mean squared error sense. And with the increase of the mulitillinearity, the new estimator is perform well.

## A numerical example

In this section, we consider a numerical example to explain the performance of theoretical result presented in “MSEM-superiority of the generalized difference-based ridge estimator $\hat{\beta }_{GRD}(k)$ over the the generalized restricted difference-based estimator $\hat{\beta }_{GRD}$” section. The data was generated by Yatchew (2003), later discussed by Tabakan and Akdeniz (2010) and came from the survey of 81 municipal electricity distribution in Ontario, Canada, in 1993.

As we all know, the partial linear model is a simple semiparametric generalization of the Cobb–Douglas model. We consider a simple variant of the Cobb–Douglas model for the cost of distributing electricity

34 $$\begin{aligned} tc=f(cust)+\beta _1wage+\beta _2pcap+\beta _3puc+\beta _4kWh+\beta _5life+\beta _6lf+\beta _7kmwire+\epsilon \end{aligned}$$

for tc stands for the log of total cost per customer, cust denotes the log of the number of customers, wage defines the log of wage rate, pcap stands for the log price of capital, puc denotes a dummy variable for the public utility commissions that deliver additional services and may benefit from economy of scope, kWh defines the log of kilowatt hours per customer, life denotes the log of the remaining life of distribution assets, lf shows the log of the load factor and kmwire presents the log of kilometers of distribution wire per customer (Tabakan and Akdeniz 2010). It is easy to see that (34) contains both nonparametric effect and parametric effects.

Since *V* is seldom known, the estimation of *V* can be used. Trenkler (1984) gave some estimates of *V* as

35 $$\begin{aligned} V=\frac{1}{\rho ^2+1}\left( \begin{array}{cccccccc} 1+\rho ^2&{}\rho &{}0&{}.&{}.&{}.&{}0&{}0\\ \rho &{}1+\rho ^2&{}\rho &{}.&{}.&{}.&{}0&{}0\\ .&{}.&{}.&{}.&{}.&{}.&{}.&{}.\\ .&{}.&{}.&{}.&{}.&{}.&{}.&{}.\\ .&{}.&{}.&{}.&{}.&{}.&{}.&{}.\\ 0&{}0&{}0&{}.&{}.&{}.&{}1+\rho ^2&{}\rho \\ 0&{}0&{}0&{}.&{}.&{}.&{}\rho &{}1+\rho ^2\\ \end{array} \right) \end{aligned}$$

where the terms of the error vector are from the MA(1) process:

$$\begin{aligned} \epsilon _i=\mu _i+\rho \mu _{i-1},\quad |\rho| <1,\quad\ i=1,2,\ldots ,n \end{aligned}$$

where $$\mu _i\sim N(0,\sigma ^2_\mu )$$, $$E(\mu _i\mu _j)=0$$, $$i\ne j$$, $$\sigma ^2=\sigma ^2_\mu (1+\rho ^2)$$.

For the linear restriction (17), the *R* is given as follows:

36 $$\begin{aligned} R=(1,-2,-2,-2,-2,-2,-2) \end{aligned}$$

In this section, we study $\rho =0.3$, $\sigma ^2_\mu =0.1$ and consider matrix *V* is estimated by (35). It is easy to compute the condition number is 2365.158, suggesting the presence of severe collinearity.

In this section we use the method which Hoerl and Kennard proposed to estimate *k*. Then we get $MSE (\hat{\beta }_{GRD}(k),\beta )=0.323$ and $MSE (\hat{\beta }_{GRD},\beta )=0.597$, that is to say the new estimator is better than restricted difference-based estimator.

Now we see theorem 21

37 $$\begin{aligned} \hat{\beta }'\left( W+\frac{2}{k}I\right) ^{-1}\hat{\beta }=0.0578<\sigma ^2 \end{aligned}$$

That is to say our numerical example satisfied with theorem 4.1. This also means our method is meaningful in practice.

## Conclusions

In this article, we present a new generalized difference-based ridge estimator that can be applied in the presence of multicollinearity in a partial linear model. Its MSE is compared analytically with the generalized restricted difference-based estimator. It is shown that for small values of the ridge parameter *k*, the new estimator is MSEM-superior to the generalized restricted difference-based estimator over an interval depending on the design points and the unknown parameter.
